# Association Between ABO Blood Type and Periodontitis: A Cross-Sectional Study

**DOI:** 10.7759/cureus.82266

**Published:** 2025-04-14

**Authors:** Mizuki Saito, Yoshihiro Shimazaki, Saori Yoshii, Tetsuhito Kojima

**Affiliations:** 1 Preventive Dentistry and Dental Public Health, Aichi Gakuin University School of Dentistry, Nagoya, JPN; 2 Dental Department, Aichi Health Promotion Foundation, Nagoya, JPN; 3 Medical Department, Aichi Health Promotion Foundation, Nagoya, JPN

**Keywords:** blood type, community periodontal index (cpi), observational cross-sectional study, oral health, periodontitis

## Abstract

Objectives: This study used health-examination data from a large population to examine the association between ABO blood type and periodontitis, considering the influence of confounding factors.

Methods:* *The study analyzed 2,311 individuals who underwent health examinations, including oral health examinations and blood-type tests. The Community Periodontal Index was used to evaluate periodontal status. Logistic regression analysis was performed with severe periodontitis as the dependent variable and blood type and other variables as independent variables; odds ratios (ORs) and 95% confidence intervals (CIs) were calculated. The sex differences in the associations between severe periodontitis and either blood type or the presence or absence of blood-type antigens (A or B antigens) were also examined.

Results: There were 879 (38.0%), 500 (21.6%), 234 (10.1%), and 698 (30.2%) participants with blood types A, B, AB, and O, respectively. Individuals with blood types A and AB had significantly higher ORs for severe periodontitis compared to those who had blood type B (OR 1.45 [95% CI 1.01-2.08] and 1.88 [95% CI 1.17-3.01], respectively). The association between blood type and severe periodontitis was significant only in men. In the association between blood-type antigen status and severe periodontitis, those with blood type A or AB who had the A antigen had a significantly higher OR for severe periodontitis than those with blood type B or O who did not have the A antigen (OR 1.40, 95% CI 1.09-1.80).

Conclusion:Individuals with blood types A and AB tended to have a higher prevalence of periodontitis. Future studies should consider the potential influence of blood type on periodontal status.

## Introduction

The best-known of the various blood types is ABO, which is categorized into four types: A, B, O, and AB. Information about ABO blood types, which were discovered by Karl Landsteiner in 1900, is considered necessary for blood and organ transplants. ABO blood types are determined by antigens on red blood cell surfaces. The antigens differ in their sugar chain structures; the H antigen is fucose added to the oligosaccharide backbone, the A antigen is N-acetylgalactosamine added to the H antigen, and the B antigen is galactose added to the H antigen. Individuals with blood type O express the H antigen, individuals with type A express the A antigen, individuals with type B express the B antigen, and individuals with type AB express both the A and B antigens.

The ABO blood type is reportedly associated with several diseases, including cancer and diabetes; the risks of these diseases vary according to blood type [1-3]. For example, *Helicobacter pylori* infections are more prevalent in type O individuals, but the risk of diabetes is lower among individuals with this blood type [3,4]. Recent studies have revealed an association between blood type and coronavirus disease 2019 (COVID-19) [5,6], and this potential relationship has received much attention.

Several studies have investigated the association between the ABO blood type and periodontal disease [7-11]. An understanding of the association between periodontal disease and blood type may facilitate periodontitis prevention through oral health instruction and dental treatment for those with high-risk blood types. However, results regarding the relationship between ABO blood type and periodontitis have been inconsistent, and no scientific consensus has been reached. Moreover, the small number of participants in most previous studies and the lack of adjustment for confounding factors reduced the credibility of the results.

This study used health-examination data from a large population to examine the association between ABO blood type, including blood-type antigen, and periodontitis, considering the influence of confounding factors. This article was previously posted to the Research Square preprint server on April 5, 2024.

## Materials and methods

Participants

This study included 2,374 individuals who underwent health examinations, including oral health examinations and blood-type tests, at the Aichi Health Promotion Foundation during the one-year period between April 1, 2018, and March 31, 2019. The Aichi Health Promotion Foundation in Nagoya, Aichi Prefecture, provides regular health examinations for corporate employees. Health examinations target the working-age population. Informed consent to use health-examination data for research was obtained from all participants. The study was approved by the Institutional Review Board of Aichi Gakuin University, School of Dentistry (approval number: 618) and conducted in strict compliance with the World Medical Association Declaration of Helsinki.

Oral health examination

The oral health examination involved evaluations of tooth condition and periodontal status. Tooth condition was assessed as the total number of sound, decayed, and filled teeth, excluding the third molars. The Community Periodontal Index (CPI) was used to evaluate periodontal status [12]. Although this method typically assesses the condition of all teeth present in the oral cavity, the standard approach used for public oral health examinations in Japan involves dividing the oral cavity into sextants and evaluating 10 representative teeth (11, 16, 17, 26, 27, 31, 36, 37, 46, and 47). Measurements were taken at six sites for each representative tooth: mesiobuccal, mid-buccal, distobuccal, distolingual, mid-lingual, and mesiolingual. The CPI scores were categorized as follows: gingival-bleeding scores were coded as absence of bleeding (code 0), presence of bleeding (code 1), or relevant tooth missing (code X). Pocket scores were coded as absence of pocket (code 0), pocket depth 4-5 mm (code 1), pocket depth ≥ 6 mm (code 2), or relevant tooth missing (code X). Twelve dentists, with calibrated inter-examiner kappa index values ≥ 0.5, examined the participants under reflected light using a dental mirror and CPI probe.

Health examination

The health examination involved height and weight measurements and blood tests. Based on the blood test results, diabetes mellitus was defined as a fasting blood glucose level of ≥ 126 mg/dL and a glycated hemoglobin (HbA1c) level of ≥ 6.5% or ongoing receipt of medical treatment for diabetes mellitus. Smoking status (never, past, or current) was assessed using a questionnaire. Blood type was determined using the antigen-antibody method.

Statistical analysis

Considering the difficulties in evaluating periodontal status among individuals with multiple tooth loss and in establishing an association between blood type and periodontitis, only participants with a current tooth count of ≥ 20 were included in the analysis. Based on the high proportion of individuals with pocket depths of 4-5 mm and the possibility that some of them had pseudopockets, the participants were classified into two groups based on maximum CPI pocket code values: those with severe periodontitis (code 2) and those without severe periodontitis (codes 0 or 1). Body mass index (kg/m2) was calculated using height and weight measurements. Regarding the association between periodontal status and each variable, analysis of variance and the chi-square test were used to assess differences in means and proportions, respectively. Univariate and multivariate logistic regression analyses were performed using the presence or absence of severe periodontitis as the dependent variable, and age, sex, body mass index, diabetes, smoking status, blood type, and number of teeth as independent variables; odds ratios (ORs) and 95% confidence intervals (CIs) were calculated. Multicollinearity between variables included in the multivariate analysis was checked using the Variance Inflation Factor statistic. The association between blood type and severe periodontitis was also analyzed by sex because the prevalence of periodontitis and lifestyle habits differ between men and women. The association between the presence or absence of a blood-type antigen (A or B antigen) and severe periodontitis was analyzed. For the A antigen, blood types A and AB were assumed to be present, and blood types B and O were considered absent. For the B antigen, blood types B and AB were assumed to be present, and blood types A and O were absent. Each blood-type antigen was analyzed separately. All statistical analyses were performed using IBM Corp. Released 2019. IBM SPSS Statistics for Windows, Version 26.0. Armonk, NY: IBM Corp. A P-value < 0.05 was considered statistically significant.

This article was written in accordance with the Strengthening the Reporting of Observational Studies in Epidemiology (STROBE) guidelines.

## Results

The analysis included 2,311 individuals, after excluding one individual with missing data and 62 individuals with fewer than 20 teeth. The age range of the participants analyzed was 23-87 years. Of these, 879 (38.0%), 500 (21.6%), 234 (10.1%), and 698 (30.2%) participants had blood types A, B, AB, and O, respectively, consistent with the blood-type distribution in the Japanese population.

The results for the association between each variable and severe periodontitis are shown in Table 1. Age, number of teeth, sex, body mass index, diabetes, smoking status, and blood type were significantly associated with severe periodontitis. Those with severe periodontitis had a greater mean age and a lower mean number of teeth. Men, those with a body mass index ≥ 25 kg/m2, those with diabetes, and those with a history of smoking had higher rates of severe periodontitis. Blood type B had the lowest proportion of individuals with severe periodontitis, followed by blood types O, A, and AB in increasing order.

**Table 1:** Associations between the characteristics of the participants and severe periodontitis SD: standard deviation

	Severe periodontitis			
Independent variable	Without (n = 1,988)	With (n = 323)		p-value
	Mean (SD)		F value	
Age (years)	48.23 (8.88)	53.79 (7.59)	113.3	< 0.001
Number of teeth	27.10 (1.50)	26.15 (2.03)	99.4	< 0.001
	n (%)			
Sex			χ^2^ value	
Men	1,444 (84.0)	276 (16.0)	24.0	< 0.001
Women	544 (92.0)	47 (8.0)		
Body mass index (kg/m^2^)				
< 18.5	131 (91.0)	13 (9.0)	17.9	< 0.001
≥ 18.5, < 25	1,336 (87.5)	190 (12.5)		
≥ 25	521 (81.3)	120 (18.7)		
Diabetes				
No	1,852 (87.3)	269 (12.7)	35.9	< 0.001
Yes	136 71.6)	54 (28.4)		
Smoking status				
Never	1,066 (92.1)	92 (7.9)	75.3	< 0.001
Past	485 (82.2)	105 (17.8)		
Current	437 (77.6)	126 (22.4)		
Blood type				
A	742 (84.4)	137 (15.6)	9.4	0.024
B	447 (89.4)	53 (10.6)		
O	606 (86.8)	92 (13.2)		
AB	193 (82.5)	41 (17.5)		

The results for the logistic regression analyses with severe periodontitis as the dependent variable are shown in Table 2. No significant multicollinearity was observed between the independent variables. Age, number of teeth, body mass index, diabetes, smoking status, and blood type were significantly associated with severe periodontitis. Those who were older, had a body mass index ≥ 25 kg/m2, had diabetes, or had a history of smoking had significantly higher ORs for severe periodontitis. Those who had many teeth or were women had significantly lower ORs for severe periodontitis. Individuals with blood types A and AB had significantly higher ORs for severe periodontitis compared with individuals who had blood type B (1.45 [95% CI 1.01-2.08] and 1.88 [95% CI 1.17-3.01], respectively).

**Table 2:** Univariate and multivariate logistic regression analyses of the associations between the independent variables and severe periodontitis OR: odds ratio; CI: confidence interval

	Dependent variable: severe periodontitis (without = 0, with = 1)			
Independent variable	Crude OR (95% CI)	p-value	Adjusted OR (95% CI)	p-value
Age (years)	1.08 (1.06-1.09)	< 0.001	1.07 (1.05-1.08)	< 0.001
Number of teeth	0.75 (0.71-0.80)	< 0.001	0.83 (0.78-0.89)	< 0.001
Sex				
Men	1		1	
Women	0.45 (0.33-0.63)	< 0.001	0.73 (0.50-1.06)	0.098
Body mass index (kg/m^2^)				
< 18.5	1		1	
≥ 18.5, < 25	0.70 (0.39-1.26)	0.232	1.07 (0.57-2.01)	0.833
≥ 25	1.62 (1.26-2.08)	< 0.001	1.31 (1.00-1.73)	0.050
Diabetes				
No	1		1	
Yes	2.73 (1.95-3.84)	< 0.001	1.51 (1.04-2.20)	0.031
Smoking status				
Never	1		1	
Past	2.51 (1.86-3.39)	< 0.001	2.00 (1.44-2.79)	< 0.001
Current	3.34 (2.50-4.47)	< 0.001	3.22 (2.32-4.46)	< 0.001
Blood type				
B	1		1	
A	1.56 (1.11-2.18)	0.010	1.45 (1.01-2.08)	0.044
O	1.28 (0.89-1.83)	0.178	1.16 (0.79-1.69)	0.452
AB	1.79 (1.15-2.79)	0.010	1.88 (1.17-3.01)	0.008

Table 3 shows the results of the logistic regression analyses of the association between blood type and severe periodontitis according to sex. This association was significant only in men. Individuals with blood type A or AB had significantly higher ORs for severe periodontitis than individuals who had blood type B (1.56 [95% CI 1.05-2.31] and 2.21 [95% CI 1.33-3.69], respectively).

**Table 3:** Logistic regression analyses of the associations between blood type and severe periodontitis according to sex *Men and women were analyzed separately. **Adjusted for age, body mass index, diabetes, smoking status, and number of teeth. OR: odds ratio; CI: confidence interval.

	Dependent variable: severe periodontitis (without = 0, with = 1)			
Independent variable*	Crude OR (95% CI)	p-value	Adjusted OR** (95% CI)	p-value
Men				
Blood type				
B	1		1	
A	1.64 (1.13-2.38)	0.009	1.56 (1.05-2.31)	0.028
O	1.21 (0.81-1.81)	0.344	1.09 (0.72-1.66)	0.689
AB	2.06 (1.27-3.33)	0.003	2.21 (1.33-3.69)	0.002
Women				
Blood type				
B	1		1	
A	1.10 (0.47-2.56)	0.831	0.97 (0.39-2.38)	0.944
O	1.57 (0.68-3.61)	0.289	1.53 (0.63-3.71)	0.351
AB	0.90 (0.27-3.05)	0.870	0.75 (0.20-2.82)	0.664

Table 4 shows the results of the logistic regression analyses of the association between blood-type antigens and severe periodontitis. Individuals with blood type A or AB who had the A antigen had a significantly higher OR (1.40 [95% CI 1.09-1.80]) for severe periodontitis than those with blood type B or O who lacked the A antigen. No significant associations were found with the presence or absence of the B antigen.

**Table 4:** Logistic regression analyses of the associations between blood-type antigen and severe periodontitis *Each blood-type antigen was analyzed separately. **Adjusted for age, sex, body mass index, diabetes, smoking status, and number of teeth. OR: odds ratio; CI: confidence interval.

	Dependent variable: severe periodontitis (without = 0, with = 1)			
Independent variable*	Crude OR (95% CI)	p-value	Adjusted OR** (95% CI)	p-value
A antigen (blood type)				
Absence (B, O)	1		1	
Presence (A, AB)	1.38 (1.09-1.75)	0.007	1.40 (1.09-1.80)	0.009
B antigen (blood type)				
Absence (A, O)	1		1	
Presence (B, AB)	0.87 (0.67-1.12)	0.269	0.96 (0.73-1.26)	0.767

## Discussion

This study found a greater prevalence of severe periodontitis in individuals with blood types A and AB, although this association was found only in men. Several studies have reported an association between blood type and periodontal disease [7-11]. A study from Turkey reported higher prevalences of gingivitis and periodontitis in individuals with blood types A and O compared with other blood types [7]. A cross-sectional study from India revealed a higher percentage of periodontitis in individuals with blood type O, and a lower percentage in those with blood type AB [9]. A meta-analysis showed that individuals with blood type AB had a lower OR for periodontitis than the ORs for other blood types [13]. By contrast, several studies have found no significant association between blood type and periodontal disease [10,11]. Thus, there are discrepancies between our results and those of previous studies. Most of the previous studies examined Indian and Turkish participants, while no study has examined Japanese participants. Therefore, the distribution of blood types among the participants varied widely among the studies. The antigens that determine blood type are secreted not only on the surface of red blood cells but also on the surface of epithelial cells and in saliva, although there are non-secreted types in which blood group antigens are not detected on epithelial cells or in saliva. About 20% of Japanese are non-secretors, and there is a difference in the risk of infectious diseases between secretors and non-secretors [14]. The percentage of non-secretors varies by blood type [15], although studies of the association between blood type and periodontal disease, including this study, have not examined non-secretors. In this study, multivariate analyses were performed using the confounding factors of age, sex, number of teeth, diabetes, and other risk factors for periodontal disease. By contrast, previous studies matched participant age groups and smoking history but did not adjust for other confounding factors [7-9]. In this study, the CPI was used as an indicator of periodontal disease, which may have led to overlooking or underestimating severe periodontitis. For these reasons, there are differences between our results and those of previous studies.

Histo-blood group antigens are suspected to play important roles in the associations between blood types and infections [16]. Pathogenic microorganisms, such as bacteria and viruses, cause infections by invading, establishing, and multiplying in host tissues. Adhesion to epithelial tissues is facilitated by ABO antigens expressed in blood cells and epithelial tissues, including tissues within the gastrointestinal tract. In particular, these antigens are expressed by the epithelium within secretory glands, such as salivary and gastric glands, leading to secretion in body fluids [17]. Certain pathogens, such as *H. pylori*, show affinity for specific ABO antigens, potentially increasing the risk of infection [18], as observed in individuals with blood type O, who are more susceptible to *H. pylori* infections, gastritis, and gastric/duodenal ulcers [2,4]. Thus, differences in pathogen binding to ABO antigens may influence the disease susceptibility associated with a particular blood type.

Periodontitis, characterized by persistent infection with periodontal pathogens [19], triggers inflammatory and immune responses in periodontal tissues through stimuli associated with periodontal pathogens. Neutrophils, interleukins 1 and 6, and tumor necrosis factor-α contribute significantly to bone resorption and tissue damage [20]. The present study found significantly higher ORs for severe periodontitis among individuals with blood types A and AB than among individuals with blood type B. Although oral bacterial status was not assessed in this study, periodontal pathogens may bind preferentially to the A antigen, facilitating tissue invasion and exacerbating periodontitis.

Individuals with blood type A have anti-B antibodies in their sera, whereas individuals with blood type B have anti-A antibodies. Individuals with blood type O possess both anti-A and anti-B antibodies, whereas individuals with blood type AB lack both antibodies. These IgM-class antibodies develop naturally without blood transfusion or pregnancy; they are potentially stimulated by intestinal microflora, such as Gram-negative bacteria, but information about their origin remains limited [21].

Natural IgM antibodies, including anti-A and anti-B antibodies, serve as the primary defense against bacterial infections through their role in activating the complement system [22]. Because A and B antigens are not exclusive to humans-they are also present in animals, and plants, and bacteria-blood-type antibodies may play a role in preventing infections [23]. Consequently, the host’s blood-type antibodies and the type and abundance of blood-group antigens in bacteria could influence an individual’s susceptibility to infections. The present study demonstrated significantly higher ORs for severe periodontitis among individuals with the A antigen than among those without the A antigen, indicating that periodontal pathogens may carry the A antigen or similar antigens. However, the binding properties of periodontal pathogens to blood-type antigens remain unknown; there is also a lack of clarity about whether bacteria carry such antigens. Further studies are needed to obtain information regarding the oral bacterial flora of participants, as well as the antigens present in the respective bacteria, to elucidate the association between blood type and periodontitis.

In this study, the association between blood type and severe periodontitis differed by sex, with significant associations found only in men. However, it is unlikely that there are sex differences in the effects of blood type. The prevalence of periodontitis has been reported to be higher in men than in women [24], and in this study, a greater percentage of men had severe periodontitis. However, there were fewer female participants, which may have made it difficult to obtain significant results.

There were several limitations in this study. Because the study focused on individuals living in a specific region of Japan, its results may not be generalizable. Considering that the distribution of blood types varies by country, different results may be observed in other countries. Additionally, as an indicator of periodontitis, the partial examination method may have underestimated periodontal status, as self-reported data on smoking were used, and reporting biases such as underreporting may have occurred. Information on nutritional status and oral health behaviors affecting periodontal status was not available in this study. Conversely, the large number of study participants and the adjustment for confounding factors were the main strengths of this study. Nevertheless, because this study lacked sufficient information to clarify the relationship between blood type and periodontitis, future studies incorporating discussions of these mechanisms are required.

## Conclusions

In this study, individuals with blood types A and AB had a higher prevalence of severe periodontitis. Assessment of conventional risk factors is crucial for preventing the onset and progression of periodontitis. Additionally, future studies should consider the potential influence of blood type on periodontal status.
